# Gastric Cancer: A Comprehensive Literature Review

**DOI:** 10.7759/cureus.55902

**Published:** 2024-03-10

**Authors:** Reda H Mithany, M Hasaan Shahid, Mina Manasseh, Muhammad Talha Saeed, Samana Aslam, Mohamed S Mohamed, Nesma Daniel

**Affiliations:** 1 Laparoscopic Colorectal Surgery, Kingston Hospital NHS Foundation Trust, Kingston Upon Thames, GBR; 2 Surgery, Glangwili General Hospital, Carmarthen, GBR; 3 General Surgery, Torbay and South Devon NHS Foundation Trust, Torquay, GBR; 4 Internal Medicine, Prince Philip Hospital, Llanelli, GBR; 5 General Surgery, Lahore General Hospital, Lahore, PAK; 6 General Surgery, Hillingdon Hospital, Uxbridge, GBR; 7 Medical Laboratory Science, Ain Shams University Specialized Hospital, Cairo, EGY

**Keywords:** gastric surgery, 5 year survival, gastrointestinal bleeding, gastric cancer, gastric neoplasia

## Abstract

Gastric cancer stands as a significant global health concern, particularly prevalent in Eastern Asia, with high mortality rates urging urgent attention and research efforts. This article comprehensively explores the epidemiology, anatomy, risk factors, pathophysiology, clinical presentation, diagnosis, staging, treatment modalities, prevention strategies, and survival rates associated with gastric cancer. Notably, *Helicobacter pylori* infection, dietary choices, and intricate stomach anatomy play pivotal roles in disease development. Early detection, utilizing staging, grading, and genetic testing for personalized treatment approaches is emphasized. Treatment modalities encompass surgery, chemotherapy, radiation therapy, targeted therapy, and immunotherapy. Prevention strategies involve lifestyle changes, screening, and genetic counseling. Survival rates vary by stage, highlighting the need for individualized care. In conclusion, a collaborative global effort is essential to address the impact of gastric cancer and improve outcomes.

## Introduction and background

A major global health concern, gastric cancer has a large impact on cancer incidence and death rates around the globe. The 2018 figures from the International Agency for Research on Cancer's GLOBOCAN project present a startling picture: more than a million new cases of stomach cancer, accounting for a sizeable share of the world's cancer burden. Remarkably, this cancer caused 8.2% of all cancer-related fatalities, highlighting its significant influence on general health. Even with notable drops in incidence, most pronounced in developed countries, stomach cancer remains a serious adversary, as it is one of the top causes of cancer-related death. China bears a sizable portion of the burden of this illness, which is primarily concentrated in Eastern and South-Eastern Asia [1,2].

There has been some progress in lessening the effects of stomach cancer, but difficulties still exist. In China, the five-year survival rate for stomach cancer is frustratingly stable at around 30%, despite notable achievements in improving survival rates, particularly through improved treatment modalities and early detection campaigns. This unyielding figure emphasizes the difficulties in preventing stomach cancer and the pressing need for ongoing study, creative thinking, and all-encompassing public health initiatives. Understanding the complex epidemiological environment and tackling the many issues raised by stomach cancer are critical as we work to combat this formidable enemy on a global scale and lessen its terrible effects on people and communities all over the world [1,2].

This review aims to provide comprehensive insights into gastric cancer, covering its epidemiology, anatomy, risk factors, pathophysiology, clinical presentation, diagnosis, staging, treatment modalities, prevention strategies, and survival rates. By offering a thorough understanding of this complex malignancy, we aim to raise awareness, encourage research efforts, and support healthcare professionals and patients in managing gastric cancer effectively.

## Review

Anatomy of the stomach

**Figure 1:**
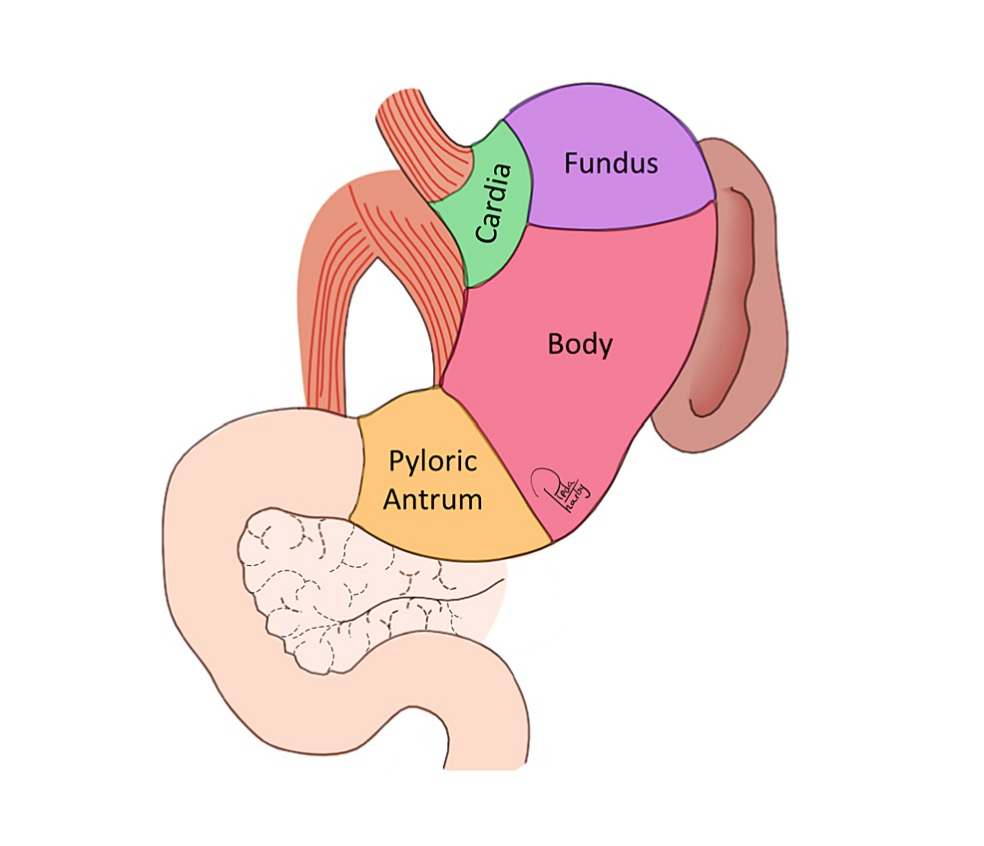
Anatomy of the stomach. Image Credit: Reda Harby (author)

The anatomical structure of the stomach holds great significance in the context of gastric cancer for several compelling reasons (Figure 1). The stomach, positioned in the upper abdominal region just below the diaphragm, fulfils a primary role as a repository for ingested food and plays a substantial role in the initial stages of the digestive process. It is noteworthy that it possesses a unique arrangement consisting of three separate muscle layers: the outer longitudinal, middle circular, and inner oblique. These muscular strata coordinate peristaltic contractions with the purpose of fragmenting digested food mechanically. The cardia connects the proximal end of the stomach to the oesophagus via a curved, J-shaped structure, whereas the pylorus delineates the distal end, which is connected to the duodenum of the small intestine [3].

This organ exhibits a remarkable capacity to regulate its volume in order to accommodate diverse dietary quantities, usually falling within 1-1.5 litres. Furthermore, a specialized mucosal layer lines the interior of the stomach and is accountable for the secretion of gastric fluids, which consist of hydrochloric acid and enzymes. The aforementioned secretions are critical for the chemical metabolism of proteins that are consumed. The complex interaction among these anatomical characteristics guarantees the efficient breakdown of food in the stomach, thus laying the foundation for subsequent digestion and nutrient assimilation in the intestines [4].

**Figure 2:**
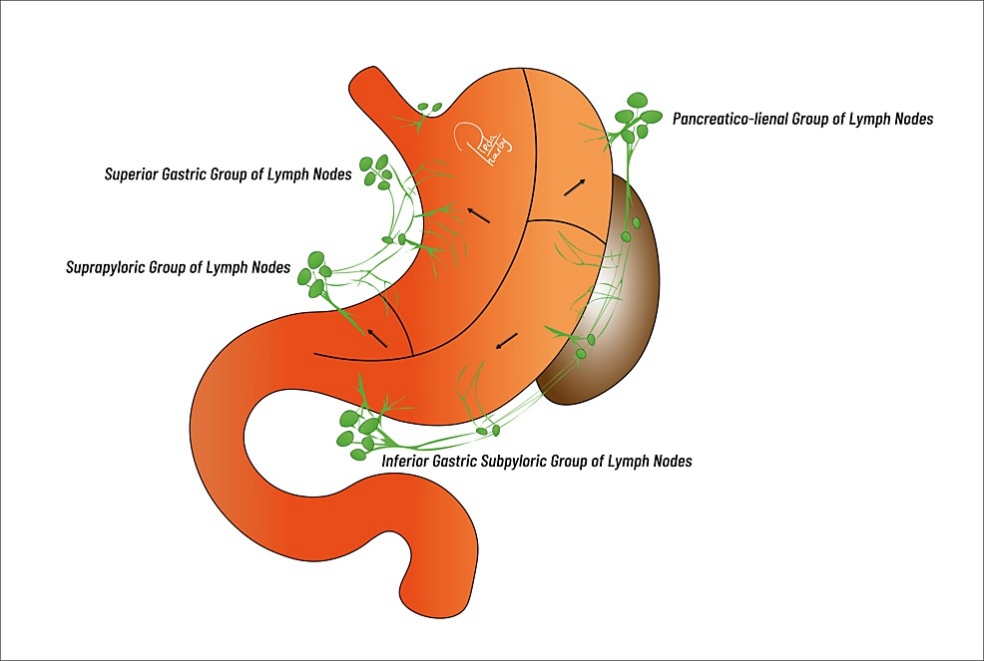
Gastric lymphatic drainage. Image Credit: Reda Harby (author)

A rich blood supply sustains the stomach, supplied by the celiac trunk. Arterial branches that contribute to vascularization include the gastroepiploic arteries, the right gastric artery, and the left gastric artery. Lymphatic drainage encompasses the perigastric nodes, hepatoduodenal ligament, splenic hilum, common hepatic artery (CHA), left gastric artery (LGA), and paraaortic drainage at its four levels (Figure 2). The stomach is supplied with innervation by the autonomic nervous system via sympathetic and parasympathetic nerves. With its left and right branches, the vagus nerve supplies vital parasympathetic innervation, whereas the sympathetic nerves utilise the celiac plexus to transmit pain signals [3,5].

The stomach wall is composed of four layers: serosa, muscularis externa, mucosa, and submucosa. Producing gastric secretions are glands located in the mucosa; the submucosa provides support for the mucosal layer. Among the three layers of the muscularis externa is the inner oblique layer, which is specifically designed to facilitate mechanical digestion. Serosa contains connective tissue that connects peritoneum to the stomach. The stomach's intricate structure facilitates the execution of its vital functions, which include digestion and nutrient processing [3].

Epidemiology and risk factors

Gastric cancer, also known as stomach cancer, is a significant global public health concern and one of the leading causes of cancer-related deaths worldwide. Its occurrence varies widely across different regions, with Eastern Asia, including countries like Japan, China, and South Korea, experiencing a disproportionately high burden, while Western countries have lower incidence rates. However, it's important to note that gastric cancer remains a substantial public health challenge even in regions with lower rates [6].

One primary risk factor for gastric cancer is chronic infection with the bacterium *Helicobacter pylori*, which colonizes the stomach lining and leads to chronic inflammation. Over time, this inflammation can transform into cancer. Dietary choices also play a significant role, with a higher risk associated with diets rich in salt-preserved foods, smoked meats, and pickled vegetables, and low in fruits and vegetables. Tobacco use and excessive alcohol consumption are established risk factors for various cancers, including gastric cancer, as they contribute to the development of precancerous lesions in the stomach lining [7,8].

Individuals with a family history of gastric cancer are at an increased risk, and specific genetic mutations, such as those related to hereditary diffuse gastric cancer syndrome (HDGC), substantially elevate the likelihood of developing gastric cancer. Prolonged inflammation of the stomach lining, as seen in conditions like chronic gastritis or pernicious anaemia, also increases the risk. Moreover, people who have undergone specific types of stomach surgery, such as partial gastrectomy for peptic ulcer disease, may face a higher risk of developing gastric cancer in the remaining stomach tissue. Gastric cancer predominantly affects older adults, and the risk of developing the disease increases with age. There is a slight male predominance in the incidence of gastric cancer [9,10].

Pathophysiology

The pathophysiology of gastric cancer is a succession of interrelated processes, constituting a complex disease. Genetic alterations in the DNA of gastric cells frequently initiate the process. These mutations can be initiated by a multitude of factors such as persistent inflammation caused by *H. pylori* infection, particular chemical exposure, or genetic predispositions. Prolonged *H. pylori* infection is frequently accompanied by chronic inflammation, which serves as a substantial predisposing factor for gastric cancer. The substance causes harm to cellular structures and promotes the proliferation of aberrant cells in the gastric mucosa. It is critical for this malignant transformation that genetic mutations accumulate, specifically in genes that regulate cell division, growth, and repair mechanisms. Oncogenes such as *HER2* and tumour suppressor genes like *TP53* are frequently impacted. Precancerous dysplastic lesions develop as a consequence of uncontrolled cellular proliferation and a diminished capacity for programmed cell death brought about by these genetic perturbations [11,12].

As the disease advances, malignant cells may acquire the capacity to invade nearby blood vessels and tissues, thereby facilitating their metastasis to adjacent lymph nodes and, ultimately, distant organs. Additionally, gastric tumours promote angiogenesis or the development of new blood vessels. This procedure guarantees an uninterrupted provision of vital nutrients and oxygen, both of which are indispensable for the proliferation and viability of malignant cells. In addition, cancer cells have the ability to evolve defence mechanisms that prevent them from being identified and attacked by the immune system. This enables them to spread and endure while evading the body's natural defences. The development of gastric cancer is also influenced by errors in the epidermal growth factor receptor (EGFR) pathway, specifically in the PI3K-AKT-mTOR signalling cascade. When PI3K is stimulated by EGF, downstream activation of AKT occurs, phosphorylating and activating mTOR. Important cellular functions like metabolism, proliferation, and survival are regulated by this system. Dysregulation causes aberrant signalling, metabolic reprogramming like the Warburg effect and uncontrolled cell growth, which are characteristics of gastric cancer. It is frequently brought on by activating mutations in members of the *EGFR* family, *PI3K*, or loss of the tumour suppressor *PTEN* [13,14].

Gastric cancer has the potential to present itself in various subtypes, each distinguished by distinct cellular characteristics and potentially pursuing idiosyncratic trajectories of development. Included in these subtypes are the intestinal and diffuse varieties. Comprehending these fundamental mechanisms is of the utmost importance in furthering our understanding of gastric cancer and formulating efficacious approaches to its detection, prevention, and management [15].

Clinical presentation and diagnosis

The clinical presentation of gastric cancer varies significantly depending on factors such as the tumor's location, size, and stage. In its early stages, gastric cancer may not show any noticeable symptoms or may present with subtle signs, while advanced-stage disease tends to produce more obvious indicators. Some common clinical signs include persistent or recurring upper abdominal discomfort or pain, often described as a burning or gnawing sensation (referred to as dyspepsia and indigestion). Profound and unexplained weight loss, often accompanied by a loss of appetite, is a common early warning sign. Chronic upper abdominal pain or discomfort, especially after meals (known as epigastric discomfort or pain), is another prevalent symptom [16,17].

Nausea, sometimes with episodes of vomiting, may occur, particularly when the tumour obstructs the passage of food through the stomach. Rapid feelings of fullness during meals, even with small amounts of food (early satiety), and difficulty swallowing (dysphagia), especially as the tumour grows and hinders food passage through the oesophagus, are additional manifestations.

Gastric cancer can also lead to gastrointestinal bleeding, resulting in signs like vomiting blood (hematemesis) or the passage of dark, tarry stools (melena). Generalized fatigue and weakness, often due to anaemia resulting from chronic blood loss, may manifest. Palpable lymph nodes in the abdomen or neck regions, indicating the potential spread of the cancer, and jaundice (yellowing of the skin and eyes) in cases where the tumour obstructs the bile duct, are further indicators [17,18].

Timely and accurate diagnosis of gastric cancer is crucial for effective treatment. The diagnostic process usually involves a thorough medical history and physical examination to assess potential risk factors and evaluate the presenting clinical symptoms. An upper gastrointestinal endoscopy allows for direct visualization of the stomach lining and the collection of tissue samples (biopsies) to confirm the presence of cancer [19].

Imaging techniques like computed tomography (CT), magnetic resonance imaging (MRI), and positron emission tomography (PET) may be used to assess the tumour's extent and the possibility of metastatic spread. Endoscopic ultrasound (EUS) provides detailed images of the gastrointestinal tract and nearby structures, aiding in the evaluation of tumour depth and lymph node involvement. Blood tests may be conducted to assess markers associated with gastric cancer, such as carcinoembryonic antigen (CEA) and carbohydrate antigen 19-9 (CA 19-9) [20,21].

In the quest for precision in gastric cancer management, molecular profiling plays a pivotal role. This involves conducting genetic testing to identify specific mutations that can guide individualized treatment strategies, thereby optimizing therapeutic outcomes. Moreover, the integration of FDA and European Medicines Agency (EMA)-approved biomarker matching targeted drugs and routine molecular pathology testing further enhances the precision of treatment approaches. For instance, a comprehensive list of gene-protein pairs, along with associated anticancer agents and indications, illustrates the diverse landscape of biomarker-driven therapies. This approach encompasses routine testing methodologies such as fluorescence in situ hybridization (FISH), immunohistochemistry (IHC), DNA sequencing, and polymerase chain reaction (PCR). By aligning these targeted drugs with specific genetic markers, clinicians can tailor treatments more effectively, marking a significant stride in advancing the management of gastric cancer [22].

Staging and grading

In the context of gastric cancer, the TNM (tumour, node, metastasis) staging system, which categorizes the extent of the tumour based on its characteristics, regional lymph node involvement, and the presence of distant metastasis, plays a pivotal role in the precise classification of the disease. Simultaneously, grading is instrumental in assessing the cellular properties and differentiation status of cancer cells, offering valuable insights into the disease's aggressiveness [23].

The TNM staging system for gastric cancer is summarized in Table 1 [24,25].

**Table 1:** Summarized Staging of Gastric Cancer Information Source: [24,25]

Stage	Tumor (T)	Lymph Nodes (N)	Metastasis (M)
0	Tis: Carcinoma in situ (limited to mucosa)	N/A	M0: No distant metastasis
I	T1: Invades lamina propria or submucosa	N0: No regional lymph node involvement	M0: No distant metastasis
	T2: Invades muscularis propria		
	T3: Invades through muscularis propria		
IIA	T1: Invades lamina propria or submucosa	N1: Involvement of 1-2 regional lymph nodes	M0: No distant metastasis
	T2: Invades muscularis propria		
	T3: Invades through muscularis propria		
IIB	T1: Invades lamina propria or submucosa	N2: Involvement of 3-6 regional lymph nodes	M0: No distant metastasis
	T2: Invades muscularis propria		
	T3: Invades through muscularis propria		
	T4a: Penetrates serosa (visceral peritoneum)		
IIC	T4a: Penetrates serosa (visceral peritoneum)	N1: Involvement of 1-2 regional lymph nodes	M0: No distant metastasis
		N2: Involvement of 3-6 regional lymph nodes	
IIIA	T4a: Penetrates serosa (visceral peritoneum)	N3: Involvement of 7 or more regional lymph nodes	M0: No distant metastasis
	T4b: Invades adjacent structures (e.g., spleen, liver)		
IIIB	T4b: Invades adjacent structures (e.g., spleen, liver)	Any N	M0: No distant metastasis
IIIC	Any T	Any N	M1: Distant metastasis (Includes peritoneal seeding; Excludes distant lymph nodes or organs

Treatment modalities

The management of gastric cancer typically involves a comprehensive approach encompassing surgery, chemotherapy, radiation therapy, and targeted therapy, immunotherapy, and combination therapies. Surgery, regarded as a primary therapeutic option, is particularly pertinent for cases characterized by localized or early-stage disease [26]. The primary goal of surgical intervention is to achieve complete resection, involving the excision of the tumor along with adjacent tissues. Established surgical techniques include gastrectomy, lymphadenectomy, and esophagogastrectomy. Additionally, specialized surgical approaches are available to address specific clinical scenarios. Minimally invasive methods, such as laparoscopy and robotic-assisted surgery, offer distinct advantages such as reduced hospitalization duration and expedited postoperative recovery [27]. Table 2 summarizes stage-wise treatment for gastric cancer according to the National Comprehensive Cancer Network (NCCN) guidelines [28].

**Table 2:** Stage-Wise Treatment For Gastric Cancer TNM: Tumor, Node, Metastasis Information Source: Chen et al., 2021 [28]

TNM Stage	Tumor Characteristics	Treatment Option
0	Tis, N0, M0	Endoscopic resection Submucosal dissection
I	T1, N0, M0	Surgical resection (partial or total)
IIA	T2, N0, M0	Surgical resection (partial or total)
IIB	T1/T2, N1, M0 T3, N0, M0	Surgical resection (partial or total) Lymphadenectomy Adjuvant chemotherapy may be considered
IIIA	T2, N2, M0 T3, N1/N2, M0	Surgical resection (partial or total) Lymphadenectomy Adjuvant chemotherapy and/or radiation
IIIB	T4a, N0/N1/N2, M0	Surgical resection (partial or total) Lymphadenectomy Adjuvant chemotherapy and/or radiation
IIIC	T4b, Any N, M0	Surgical resection (partial or total) Lymphadenectomy Adjuvant chemotherapy and/or radiation
IV	Any T, Any N, M1	Adjuvant chemotherapy and/or radiation Palliative treatments (chemo, targeted therapy, immunotherapy) Surgery for symptom relief or complications Clinical trials for experimental treatments

Subtotal gastrectomy is targeted at tumors in the lower stomach, aiming to preserve upper digestive functionality, while total gastrectomy is indicated for larger or upper stomach tumors, necessitating substantial dietary adjustments post surgery [29]. Palliative surgery is considered for advanced, non-curative cases, with the primary aim of alleviating symptoms and complications. In cases of peritoneal dissemination, cytoreductive surgery combined with hyperthermic intraperitoneal chemotherapy (HIPEC) may be contemplated. Endoscopic resection is an appropriate option for select early-stage cases, facilitating minimally invasive tumor removal. A multidisciplinary approach is imperative, involving collaboration among surgeons, oncologists, and gastroenterologists. This collaborative effort is essential to customize the surgical strategy in accordance with the distinctive attributes of gastric cancer and the patient's individual medical history [30].

Chemotherapy, constituting the use of pharmacological agents to exterminate or impede the growth of cancer cells, stands as another integral facet of gastric cancer treatment. It may be administered in the neoadjuvant setting to reduce tumor size prior to surgery, in the adjuvant context to eliminate any residual cancer cells following surgery, or as the primary therapeutic modality for advanced or metastatic cases. The integration of chemotherapy, either prior to or subsequent to surgical intervention, augments the prospects of survival in patients diagnosed with stage 1B or higher gastric cancers [31].

Radiation therapy, employing high-energy beams to specifically target and annihilate cancer cells, holds a distinct place in the therapeutic armamentarium. It may be applied preoperatively (neoadjuvant) to reduce tumor size or postoperatively (adjuvant) to eliminate any residual cancer cells. In some instances, radiation therapy is harnessed to alleviate symptoms or manage advanced disease. The efficacy of radiation therapy in mitigating symptoms associated with advanced gastric cancer is well-documented. Furthermore, stereotactic body radiotherapy (SBRT) exhibits potential in extending disease control for a subgroup of gastric cancer patients bearing limited metastases in the liver [32,33].

Targeted therapy entails the utilization of medications designed to specifically target molecular aberrations or markers present in cancer cells. In the context of gastric cancer, drugs such as trastuzumab (Herceptin) and ramucirumab (Cyramza) have been developed to target particular proteins implicated in cancer growth and progression. The amalgamation of trastuzumab, ramucirumab, and paclitaxel has exhibited noteworthy efficacy alongside tolerable safety profiles, particularly in individuals who have previously undergone treatment for HER2-positive gastric or gastroesophageal junction (G/GEJ) cancer [34].

Immunotherapy revolutionizes cancer treatment by activating the immune system against cancer cells. Key drugs like pembrolizumab and nivolumab show promise in specific gastric cancer types. These immune checkpoint inhibitors (ICIs) have become standard in treating advanced gastric cancer, but not all patients respond well, highlighting the need for better patient selection and combination therapies. Three anti-PD-1 antibodies are approved by FDA in different regions, reflecting the evolving landscape of immunotherapy [35]. Novel treatments for gastric cancer are now being investigated in a number of active clinical trials. Among these is a phase III trial called MK-3475-062, or KEYNOTE-062, which compares the checkpoint inhibitor pembrolizumab to a placebo in patients with stomach or gastroesophageal junction cancer. The purpose of this clinical trial is to assess the effectiveness of pembrolizumab in terms of overall survival and progression-free survival. An additional important trial, called DESTINY-Gastric02, is assessing patients with gastric or gastroesophageal junction adenocarcinomas that overexpress HER2 with trastuzumab deruxtecan (T-DXd), an antibody-drug combination targeting HER2. The purpose of this trial is to ascertain the participants' progression-free survival, overall response rate, and length of response to T-DXd. These trials give promise for better outcomes for people dealing with this difficult disease and represent major advances in the hunt for efficient treatments for gastric cancer [36,37].

Prevention strategies

Gastric cancer prevention strategies center on minimizing risk factors and adopting lifestyle changes to reduce the likelihood of disease development. Key approaches include the eradication of *H. pylori *infection, a significant risk factor, through a combination of antibiotics and acid-reducing medications. A balanced and nutritious diet, emphasizing fruits, vegetables, and lean proteins while limiting high-salt, processed foods, and red meats, contributes to prevention. Additionally, quitting smoking and moderating alcohol intake are crucial in reducing the associated cancer risk. Regular physical activity, maintaining a healthy body weight through a combination of diet and exercise, and limiting exposure to known carcinogens further contribute to prevention efforts. Screening and early detection are emphasized, particularly for individuals at heightened risk, utilizing endoscopic examinations and biopsies. Genetic counseling and testing are recommended for those with a family history or genetic syndromes linked to an increased risk. Promoting a diverse gut microbiome through probiotics and a fiber-rich diet may also play a role in reducing gastric cancer risk. Lastly, routine health check-ups with healthcare providers are essential for monitoring overall health and identifying potential risk factors or early signs of gastric cancer [38-40].

Survival rates

Survival rates associated with gastric cancer exhibit considerable variation contingent on several determinants, encompassing the stage at diagnosis, the tumor's anatomical location, and the patient's overall health. Survival rates are customarily expressed as the percentage of individuals who survive for a specified period following diagnosis. It is essential to acknowledge that survival rates represent estimates founded on historical data and may not necessarily reflect individual outcomes. Table 3 summarizes stage wise five-year survival rate of gastric cancer [41,42].

**Table 3:** Stage-Wise Five-Year Survival Rate Information Source: [41,42]

Stage	Description	5-Year Survival Rate
Stage 0	Tis, N0, M0 (Very early, localized)	90-95%
Stage I	T1-2, N0, M0 (Localized, deeper infiltration)	70-85%
Stage II	T1-2, N1-2, M0 or T3, N0-1, M0 (Localized, lymph node involvement)	30-70%
Stage III	T4, N0-2, M0 or T1-3, N2-3, M0 (More advanced, significant lymph node involvement)	20-40%
Stage IV	Any T, Any N, M1 (Metastatic, distant spread)	Less than 5%

Advancements in treatment modalities, early detection methods, and enhancements in supportive care can collectively influence survival rates. Moreover, the specific subtype of gastric cancer, the presence of particular genetic mutations, and the patient's responsiveness to treatment can exert a substantial influence on survival outcomes.

## Conclusions

Gastric cancer poses a formidable global health challenge, particularly in Eastern Asia, marked by high mortality rates. This comprehensive review covers epidemiology, emphasizing *H. pylori* infection and dietary choices as pivotal risk factors. The intricate anatomy of the stomach plays a crucial role in disease localization, staging, and treatment planning, with genetic mutations and inflammation central to the pathophysiology. Early detection is underscored through clinical presentation and diagnosis processes, utilizing staging, grading, and genetic testing for personalized treatment approaches, including surgery, chemotherapy, radiation therapy, targeted therapy, and immunotherapy. Prevention strategies emphasize lifestyle changes and screening, while survival rates vary by stage, underscoring the need for individualized care. In conclusion, addressing gastric cancer requires a collaborative effort to reduce its impact and enhance outcomes on a global scale.
